# Intimate partner violence (IPV): The validity of an IPV screening instrument utilized among pregnant women in Tanzania and Vietnam

**DOI:** 10.1371/journal.pone.0190856

**Published:** 2018-02-01

**Authors:** Vibeke Rasch, Toan Ngo Van, Hanh Thi Thuy Nguyen, Rachel Manongi, Declare Mushi, Dan W. Meyrowitsch, Tine Gammeltoft, Chun Sen Wu

**Affiliations:** 1 Department of Obstetrics and Gynecology, Odense University Hospital, Odense, Denmark; 2 Department of Clinical Research, University of Southern Denmark, Odense, Denmark; 3 Institute of Preventive Medicine and Public Health, Hanoi Medical University, Hanoi, Vietnam; 4 Department of Public Health, Kilimanjaro Christian Medical University College, Moshi, Tanzania; 5 Department of Public Health, University of Copenhagen, Copenhagen, Denmark; 6 Department of Anthropology, University of Copenhagen, Copenhagen, Denmark; Royal Children’s Hospital, AUSTRALIA

## Abstract

**Background:**

Intimate partner violence (IPV) is a global problem that affects one-third of all women. The present study aims to develop and determine the validity of a screening instrument for the detection of IPV in pregnant women in Tanzania and Vietnam and to determine the minimum number of questions needed to identify IPV.

**Method:**

An IPV screening instrument based on eight questions was tested on 1,116 Tanzanian and 1,309 Vietnamese women who attended antenatal care before 24 gestational weeks. The women were re-interviewed during their 30^th^-34^th^ gestational week where the World Health Organization (WHO) IPV questionnaire was used as the gold standard. In all, 255 combinations of eight different questions were first tested on the Tanzanian study population where sensitivity, specificity, positive predictive value, negative predictive value and accuracy were calculated. In the evaluation of the performance of the question combinations, different IPV types and the frequency of abusive acts were considered. The question combinations that performed best in Tanzania were subsequently evaluated in the Vietnamese study population.

**Results:**

In Tanzania, a combination of three selected questions including one question on emotional IPV, one on physical IPV and one on sexual IPV was found to be most effective in identifying women who are exposed to at least one type of IPV during pregnancy (sensitivity = .80; specificity = .74). The performance of the identified combination was slightly less effective in Vietnam (sensitivity = .74; specificity = .68). Focusing on different IPV types, the best performance was found for exposure to physical IPV in both Tanzania (sensitivity = .93; specificity = .70) and Vietnam (sensitivity = .96; specificity = .55). In both countries, the sensitivity increased with the frequency of abuse whereas the specificity decreased.

**Conclusion:**

By asking pregnant women three simple questions we were able to identify women who were exposed to IPV during pregnancy in two different countries. The question combination performed best in assessing physical IPV where it identified 93% and 96% of Vietnamese and Tanzanian women, respectively, who were exposed to physical IPV.

## Introduction

Intimate partner violence (IPV) is a worldwide problem, which globally affects one-third of all women [1–4]. IPV during pregnancy is associated with an increased risk of adverse pregnancy outcomes [5]. We have previously documented that pregnant women in Tanzania and Vietnam exposed to IPV have a three to six times increased risk of preterm birth and low birth weight [3,4].

Addressing the problem of IPV is an international priority [2] and consequently a number of guidelines on how to deal with IPV have been published [6,7]. Implicit in these guidelines is the assumption that identification of women who are exposed to violence will be enhanced by screening. Therefore different research measures, including the WHO Multi-country Study Instrument [8], have been developed to identify women who are exposed to IPV. However, WHO do not support screening in settings where onwards referrals and appropriate training are not in place. In 2010, Centers for Disease Control and Prevention made a compendium of these measures to help health care providers addressing IPV [9].

In the implementation of any screening programme, the validity of the screening instruments should be considered. According to a systematic review, there is a great variation in sensitivity (0.30–1.00) and specificity (0.55–0.99) of different IPV screening instruments used in health care settings [10]. There is no clear definition of the minimum standards of a screening instrument. It has, however, been suggested that clinicians and policy makers ideally should select screening instruments that have both sensitivity and specificity greater than 0.80 [11]. The cultural transferability should further be taken into account, since tools may perform differently according to the setting in which they have been developed. This aspect has been stressed in a recent systematic review which found that over 70% of the included studies were conducted in America [12]. It may thus be argued that in an international context there is a need of evaluating and testing screening instruments in more diverse settings.

According to a recent Cochrane review, screening of women who attend antenatal care may be helpful in identifying women who are exposed to IPV [13]. Similar findings have been reported in a scoping review of IPV screening programs [14]. However, the reviews do also stress that there is still a paucity of high quality evidence to support routine IPV screening. This may especially apply for settings where there is not a well-developed service response for women who are exposed to IPV. Although these reservations prevail, it may be argued that the high prevalence rate of IPV among pregnant women and the well-known negative impact it has on pregnancy outcome provides a strong rationale for optimizing health care settings to identify pregnant women who are exposed to IPV and initiate IPV services. This notion is supported by the fact that more than 80% of pregnant women in low- and middle-income countries (L/MIC) attend antenatal care at least once [15]. It may therefore be argued that antenatal care comprises a unique opportunity to identify pregnant women who are exposed to IPV in L/MIC. However, the time and resource constraints imposed on the health staff has to be considered. Therefore the identification of women who are exposed to IPV should rely on brief screening tools, which have undergone country specific validation.

Although IPV during pregnancy is a major health problem in L/MIC, with severe adverse health consequences, few screening programmes have been evaluated and implemented in such settings [12]. Against this background, the present study aims to develop and test a simple, brief screening instrument for the detection of IPV in pregnant women in Tanzania and Vietnam, taking into account the different IPV types and the frequency of abusive acts.

## Material and methods

The present study was part of a larger research project, "The Impact of Violence on Reproductive Health in Tanzania and Vietnam (PAVE)", with the overall aim of assessing the associations between IPV and adverse pregnancy outcomes and antenatal depression in Tanzania and Vietnam. The study was performed simultaneously in the two countries. In Tanzania, women were recruited from Majengo Health Centre and Pasua Health Centre, in Moshi District, and in Vietnam they were recruited from Dong Anh Hospital and Bac Thang Long Hospital in Dong Anh District. A total of 1,123 Tanzanian women and 1,337 Vietnamese women who attended antenatal care before the 24^th^ gestational week were enrolled. In both settings, the women were interviewed when included in the study, at 30–34 gestational weeks, at delivery, and 4–12 weeks postpartum. At enrolment, information on socioeconomic and reproductive characteristics was obtained and an agreement was reached regarding time and place for the second interview. In all, 1,116 Tanzanian women and 1,309 Vietnamese women were re-interviewed in gestational week 30–34. The second interview, which was performed in homes or similar places where participants felt comfortable to talk freely, included detailed information on the women’s exposure to IPV.

### Screening instrument and gold standard

**Fig 1:**
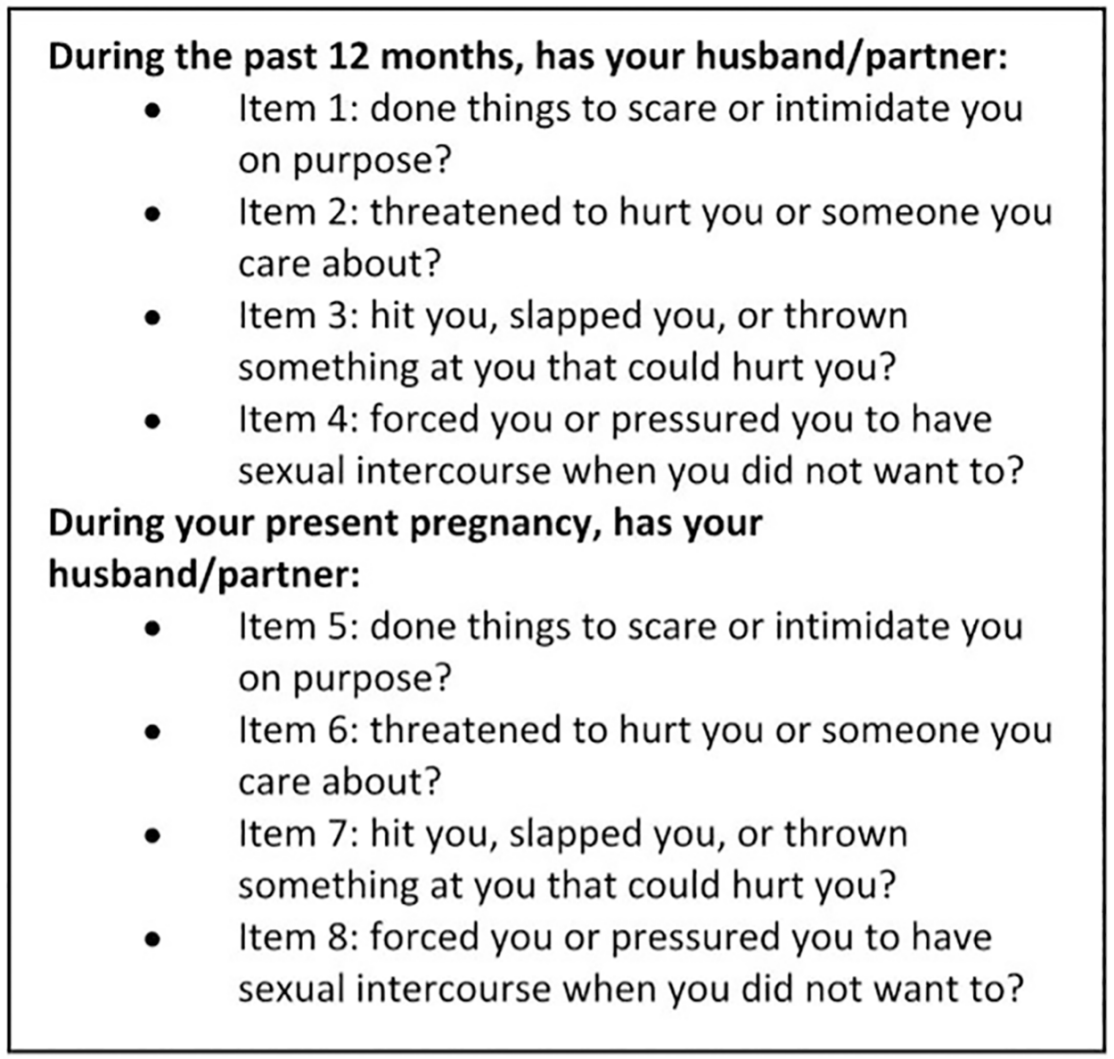
Screening questions.

Different questionnaires and screening tools [8,9,16] were assessed for their appropriateness in being used to identify pregnant women in Tanzania and Vietnam who are exposed to IPV. After the assessment we decided to use four questions on exposure to emotional, physical and sexual IPV from the WHO questionnaire and included these in the inclusion interview. Some minor modifications were done regarding recall period for experience of IPV where we focused on “during the past 12 months” and “during this pregnancy”. The phrasings of the questions were: In the past 12 months, has your husband/partner: 1) Done things to scare or intimidate you on purpose? y/n; 2) Threatened to hurt you or someone you care about? y/n; 3) Hit you, slapped you, or thrown something at you that could hurt you? y/n; 4) Forced you or pressured you to have sexual intercourse when you did not want to? y/n. The woman was subsequently asked the same four questions in relation to her current pregnancy. The women were thus asked 8 questions in all, focusing on IPV exposure 12 months before and during the present pregnancy (Fig 1). The questions were translated from English to Kiswahili by one native Kiswahili speaker and from English to Vietnamese by one native Vietnamese speaker.

To investigate the validity of the 8 questions (4 questions on IPV exposure during the past 12 months and 4 questions on IPV exposure during pregnancy), the pregnant women were re-interviewed in gestational week 30–34. At the re-interview, the full WHO questionnaire for assessing Domestic Violence against Women was applied and used as a “gold standard”. The WHO questionnaire comprises 5 questions on emotional IPV, 6 questions on physical IPV and 4 questions on sexual IPV. For each question, the women are asked if they ever have been exposed to IPV and if yes, if they have been exposed during the past 12 months, and if yes, whether during the past 12 months it has happened once, a few times or many times. Since the study aimed at assessing exposure to IPV during pregnancy, the assessment period of “past 12 months” in the original instrument was changed to “during this pregnancy” and to operationalize the frequency of IPV exposure, “a few times” was defined as 2–5 times and “many times” as more than 5 times.

### Data analysis

All data were double-entered in Epi Data (Version 3.1) by two different data clerks, and discrepancies were identified and subsequently corrected according to the original data forms. All statistical analyses were performed using the STATA software package (version 14).

To determine the properties of the eight questions for detecting exposure to at least one type of IPV (regardless of type), emotional IPV, physical IPV, and sexual IPV, the following values were determined: 1) sensitivity (proportion of women exposed to IPV who screened positive), 2) specificity (proportion of women not exposed to IPV who screened negative), 3) positive predictive value (PPV; proportion of all positively screened women who were truly exposed to IPV), 4) negative predictive value (NPV; proportion of all negatively screened women who were truly not exposed to IPV) and 5) accuracy. In all, 255 question combinations were generated for each type of IPV. The minimum score possible was zero (the woman replied no to all eight questions) and the maximum score possible was eight (the woman replied yes to all eight questions). To select the optimal cut-off score, the properties of the question combinations were computed according to different cut-off levels (cut-off 1–8).

The questions’ performance was first tested on the Tanzanian study population. In the evaluation of the different combinations of the questions, we first gave priority to their properties in predicting *at least one type of IPV* and selected the combinations with a sensitivity > 0.75, an NPV > 0.75, and accuracy > 0.75 when assessed at a cut-off level of 1. This resulted in 144 combinations, which were subsequently assessed for their properties in predicting *emotional IPV*, *physical IPV* and *sexual IPV* with an accuracy > 0.70. The question combinations were subsequently evaluated for their properties in predicting exposure to the different forms of IPV according to the frequency of exposure; only once, 2–5 times and more than 5 times.

The combinations that performed best in identifying the different forms of IPV (at least one type of IPV, emotional, physical or sexual IPV) were thereafter examined according to the cut-off value which resulted in highest sensitivity and specificity. Then, it was observed whether this cut-off also yielded the highest accuracy.

Finally, the combinations that were found to perform best in Tanzania were evaluated on the Vietnamese study population [17,18] for their ability to detect at least one type of IPV as well as their properties in predicting *emotional IPV*, *physical IPV* and *sexual IPV*. The diagnostic ability of the question combination found to have the highest sensitivity and specificity for determining all four types of IPV was tested for different cut-off values in the Vietnamese study population as well.

### Ethical considerations

Female nurses and community collaborators were recruited as research assistants. They received comprehensive training on the concepts of gender, gender discrimination, inequality, domestic violence and how to deal with participants who were exposed to violence. The WHO Ethical and Safety Recommendations for Research on Domestic Violence against Women [19] was used to guide this training. During proposal development and as part of the project work, meetings with relevant health providers, the legal authorities, the police, women’s organizations and religious organizations were arranged. Through these meetings, IPV counsellors and service organizations were identified and their contact information was noted on a list that was made available at the antenatal clinics’ note boards. The training of research assistants emphasized how they should interact with women who were exposed to violence. The research assistants were instructed to assist the women if they were asked but it was stressed that they should not tell the exposed women what to do or make it a personal responsibility to assure that the violence came to an end. If the women during the interviews revealed that they were exposed to on-going physical and/or sexual violence they were provided with a “hard referral” where a separate counsellor, who was linked to the research, referred women to specific services after receiving the participant’s consent.

All women were informed in detail about the study and written informed consent was obtained. In Tanzania, none of the participants were aged below 18 years. In Vietnam, two were aged 17 and accompanied by their mother, who also signed the consent form. Ethical approval of the study was obtained from the Ethical Review Committee at Kilimanjaro Christian Medical University and Hanoi Medical University.

## Results

In Tanzania, a total of 1,123 women met the inclusion criteria. As seven women did not show up for IPV assessment in gestational week 30–34, the final sample comprised of 1,116 women. In Vietnam, 1,337 women fulfilled the inclusion criteria and 1,309 presented for IPV assessment in gestational week 30–34.

### Socio-economic characteristics and exposure to IPV in Tanzania and Vietnam

Socio-economic characteristics and ever-exposure to IPV among Tanzanian and Vietnamese women are listed in Table 1. The Tanzanian and Vietnamese women differed from each other regarding age, where Tanzanian women were younger; parity, where Tanzanian women were of higher parity; education, where Vietnamese women had been schooling for more years; and employment, where Vietnamese women were more often employed. Vietnamese women had more often ever been exposed to any emotional IPV (57% vs 31%) and physical IPV (13% vs 11%). In contrast Tanzanian women had more often been exposed to sexual IPV (19% vs 13).

**Table 1:** Socioeconomic characteristics and IPV exposure ever of the study population in Tanzania and Vietnam.

	Tanzania (n = 1,116)		Vietnam (n = 1,309)	
	N	%	N	%
Age				
17–19	143	12.8	25	1.9
20–24	376	33.7	432	33.0
25–29	316	27.8	485	37.1
30–34	166	14.9	261	19.9
35+	121	10.8	106	8.1
No. of children				
0	429	38.4	592	45.2
1–2	533	47.8	699	53.4
3+	154	13.8	18	1.4
Years schooling				
<7 years	676	60.6	49	3.7
8–11 years	390	34.9	221	16.9
12+ years	50	4.5	1039	79.4
Occupation				
Employed	179	16.0	425	32.5
Self-employed	512	45.9	805	61.5
Unemployed	425	38.1	79	6.0
IPV exposure ever				
At least one type of IPV	438	39.2	766	58.5
Emotional IPV	344	30.8	743	56.7
Physical IPV	119	10.7	169	12.9
Sexual IPV	215	19.3	166	12.7

When focusing on exposure to IPV during pregnancy, almost one-third of the Tanzanian women had been exposed to at least one type of IPV during their current pregnancy, 23% had been exposed to emotional IPV, 6.0% to physical IPV and 15% to sexual IPV (Table 2). The vast majority (76–90%) of the exposed women reported that they had been abused 2–5 times, whereas 6–10% reported they had been abused more than 5 times. In Vietnam more than one-third (35%) had been exposed to at least one type of IPV, 32% had been exposed to emotional IPV, 3.5% to physical IPV and 10% to sexual IPV. In comparison with Tanzanian women, Vietnamese women reported 2–5 times exposure less frequently (33–72%) and exposure of more than 5 times more frequently (7–24%).

**Table 2:** Exposure to at least one type of IPV (emotional, physical and sexual IPV) during pregnancy based on WHO standards.

	Tanzania (n = 1,116)		Vietnam (n = 1,309)	
	N	%	N	%
At least on type of IPV during pregnancy	337	30.2	461	35.2
Once	20	5.9	65	14.1
2–5 times	293	86.9	325	70.5
More than 5 times	24	7.1	71	15.4
Emotional IPV during pregnancy	254	22.8	421	32.2
Once	12	4.7	66	15.7
2–5 times	216	85.0	303	72.0
More than 5 times	26	10.2	52	12.3
Physical IPV during pregnancy	67	6.0	46	3.5
Once	11	16.4	28	60.9
2–5 times	51	76.1	15	32.6
More than 5 times	5	7.5	3	6.5
Sexual IPV during pregnancy	172	15.4	130	9.9
Once	8	4.7	10	7.7
2–5 times	154	89.5	89	58.5
More than 5 times	10	5.8	31	23.8

### Effectiveness of the screening questions for the assessment of at least one type of IPV (physical, emotional and sexual IPV)

The psychometric properties for detecting at least one type of IPV (physical, emotional and sexual IPV), using the WHO questionnaire as the “gold standard” were tested for all 255 combinations of the eight questions among Tanzanian women. Four combinations: 1+4, 1+2+4, 1+3+4, and 1+2+3+4 were found to have sensitivity > 0.75, NPV > 0.75 and accuracy > 0.70 for predicting at least one type of IPV (emotional, physical and sexual IPV) (Table 3). Question combination 1+3+4 was found to have the most favourable psychometric properties for detecting at least one type of IPV (sensitivity = .80; specificity = .86); emotional IPV (sensitivity = .78; specificity = .79); physical IPV (sensitivity = .93; specificity = .70); and sexual IPV (sensitivity = .87; specificity = .76). This item combination also yielded an acceptable NPV and accuracy, varying from 0.91–0.99 and 0.71–0.84, respectively. If focusing on identifying repeated IPV, the sensitivity of question combination 1+3+4 increased slightly whereas the specificity decreased. More specifically, the following psychometric properties/characteristics were found: 2–5 times exposure to at least one type of IPV (sensitivity = .81; specificity = .84); 2–5 times exposure to emotional IPV (sensitivity = .80; specificity = .77); 2–5 times exposure to physical IPV (sensitivity = .96; specificity = .69); and 2–5 times exposure to sexual IPV (sensitivity = .87; specificity = .74) with NPV and accuracy varying from 0.92–1.00 and 0.70–0.83, respectively. When the screening tool was only applied on women who had been exposed to IPV more than 5 times, the sensitivity for detecting physical and sexual IPV increased to 1.00 and 0.90 whereas it decreased for detecting at least one type of IPV and emotional IPV; the accuracy fell to 0.66–0.68, reflecting the relatively few women who had been exposed to IPV more than five times during pregnancy. The selected item combinations were subsequently tested on the Vietnamese study population, where the performance was comparatively poorer (Table 4). However, question combination 1+3+4 remained the combination that had the best psychometric properties for detecting at least one type of IPV (sensitivity = 0.74; specificity = 0.68); emotional IPV (sensitivity = 0.76; specificity = 0.67); physical IPV (sensitivity = 0.96; specificity = 0.55); and sexual IPV (sensitivity = 0.71; specificity = 0.56). Similarly, both NPV and accuracy were comparatively lower in the Vietnamese population, varying from 0.83–1.00 and 0.56–0.70, respectively. When focusing on identification of repeated IPV, the sensitivity and specificity remained almost the same, with values varying from 0.71–0.93 and 0.54–0.64, respectively, for women who had been exposed to IPV 2–5 times and values varying from 0.67–0.86 and 0.53–0.56, respectively, for women who had been exposed to IPV more than 5 times.

**Table 3:** Sensitivity (Sens), specificity (Spe), negative predictive value (NPV) and accuracy (Acc) of the selected questions combinations—Tanzania.

		1 time exposure				2–5 times exposure				More than 5 times exposure			
		Sens	Spe	NPV	Acc	Sens	Spe	NPV	Acc	Sens	Spe	NPV	Acc
*At least one type of IPV*	Question(1+4)	0.78	0.86	0.9	0.84	0.79	0.85	0.91	0.83	0.74	0.68	0.99	0.68
	Questions(1+2+4)	0.79	0.86	0.9	0.84	0.81	0.84	0.92	0.83	0.76	0.67	0.99	0.68
	Questions(1+3+4)	0.80	0.86	0.91	0.84	0.81	0.84	0.92	0.83	0.76	0.67	0.99	0.67
	Questions(1+2+3+4)	0.80	0.85	0.91	0.84	0.82	0.84	0.92	0.83	0.76	0.67	0.99	0.67
*Emotional IPV*	Question(1+4)	0.76	0.79	0.92	0.79	0.79	0.78	0.94	0.78	0.69			
	Questions(1+2+4)	0.78	0.79	0.92	0.79	0.80	0.78	0.94	0.78	0.73			
	Questions(1+3+4)	0.78	0.79	0.92	0.78	0.80	0.77	0.94	0.78	0.73			
	Questions(1+2+3+4)	0.78	0.78	0.92	0.78	0.81	0.77	0.94	0.78	0.73			
*Physical IPV*	Question(1+4)	0.85	0.70	0.99	0.71	0.87	0.69	0.99	0.7	1	0.67	1	0.67
	Questions(1+2+4)	0.88	0.69	0.99	0.71	0.91	0.69	0.99	0.7	1	0.66	1	0.66
	Questions(1+3+4)	0.93	0.70	0.99	0.71	0.96	0.69	1	0.7	1	0.66	1	0.66
	Questions(1+2+3+4)	0.93	0.69	0.99	0.71	0.96	0.68	1	0.7	1	0.66	1	0.66
*Sexual IPV*	Question(1+4)	0.87	0.76	0.97	0.78	0.86	0.75	0.97	0.77	0.90	0.67	1	0.67
	Questions(1+2+4)	0.87	0.76	0.97	0.78	0.87	0.75	0.97	0.76	0.90	0.67	1	0.67
	Questions(1+3+4)	0.87	0.76	0.97	0.77	0.87	0.74	0.97	0.76	0.90	0.66	1	0.67
	Questions(1+2+3+4)	0.87	0.75	0.97	0.77	0.87	0.74	0.97	0.76	0.90	0.66	1	0.66

Question 1: Done things to scare or intimidate you on purpose?

Question 2: Threatened to hurt you or someone you care about?

Question 3: Hit you, slapped you, or thrown something at you that could hurt you?

Question 4: Forced you or pressured you to have sexual intercourse when you did not want to?

**Table 4:** Sensitivity (Sens), specificity (Spe), negative predictive value (NPV) and accuracy (Acc) of the selected questions combinations, Vietnam.

		1 time exposure				2–5 times exposure				More than 5 times exposure			
		Sens	Spe	NPV	Acc	Sens	Spe	NPV	Acc	Sens	Spe	NPV	Acc
*At least one type of IPV*	Question(1+4)	0.73	0.69	0.82	0.71	0.73	0.64	0.87	0.67	0.78	0.56	0.98	0.57
	Questions (1+2+4)	0.73	0.69	0.82	0.71	0.73	0.64	0.87	0.67	0.78	0.56	0.98	0.57
	Questions (1+3+4)	0.74	0.68	0.83	0.70	0.74	0.64	0.87	0.66	0.8	0.55	0.98	0.56
	Questions(1+2+3+4)	0.74	0.68	0.83	0.70	0.74	0.64	0.87	0.66	0.8	0.55	0.98	0.56
*Emotional IPV*	Question(1+4)	0.75	0.68	0.85	0.70	0.74	0.63	0.89	0.66	0.84			
	Questions (1+2+4)	0.75	0.68	0.85	0.70	0.74	0.63	0.89	0.66	0.84			
	Questions (1+3+4)	0.76	0.67	0.85	0.70	0.76	0.62	0.89	0.66	0.86			
	Questions(1+2+3+4)	0.76	0.67	0.85	0.70	0.76	0.62	0.89	0.66	0.86			
*Physical IPV*	Question(1+4)	0.91	0.56	0.99	0.57	0.93	0.55	1	0.55	1	0.54	1	0.54
	Questions (1+2+4)	0.91	0.56	0.99	0.57	0.93	0.55	1	0.55	1	0.54	1	0.54
	Questions (1+3+4)	0.96	0.55	1	0.56	0.93	0.54	1	0.54	1	0.53	1	0.53
	Questions(1+2+3+4)	0.96	0.55	1	0.56	0.93	0.54	1	0.54	1	0.53	1	0.53
*Sexual IPV*	Question(1+4)	0.71	0.57	0.95	0.58	0.71	0.56	0.96	0.57	0.67	0.55	0.99	0.55
	Questions (1+2+4)	0.71	0.57	0.95	0.58	0.71	0.56	0.96	0.57	0.67	0.55	0.99	0.55
	Questions (1+3+4)	0.71	0.56	0.95	0.57	0.71	0.55	0.96	0.56	0.67	0.54	0.99	0.54
	Questions(1+2+3+4)	0.71	0.56	0.95	0.57	0.71	0.55	0.96	0.56	0.67	0.54	0.99	0.54

Question 1: Done things to scare or intimidate you on purpose?

Question 2: Threatened to hurt you or someone you care about?

Question 3: Hit you, slapped you, or thrown something at you that could hurt you?

Question 4: Forced you or pressured you to have sexual intercourse when you did not want to?

### Cut-off values and psychometric properties

**Fig 2:**
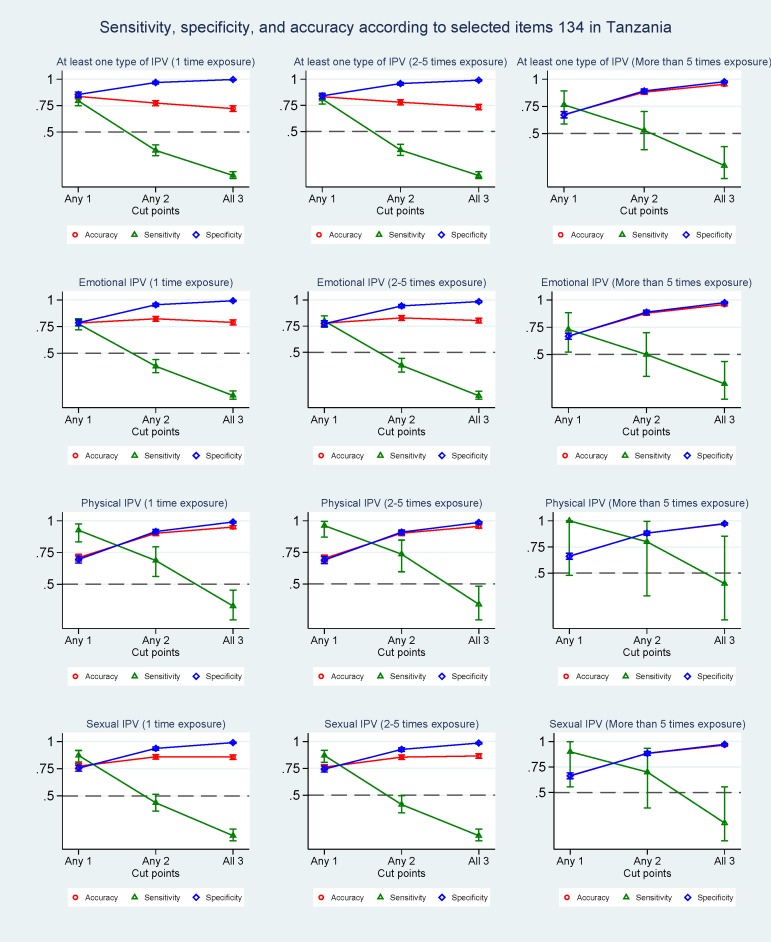
Sensitivity, specificity, and accuracy according to selected items 134 in Tanzania.

**Fig 3:**
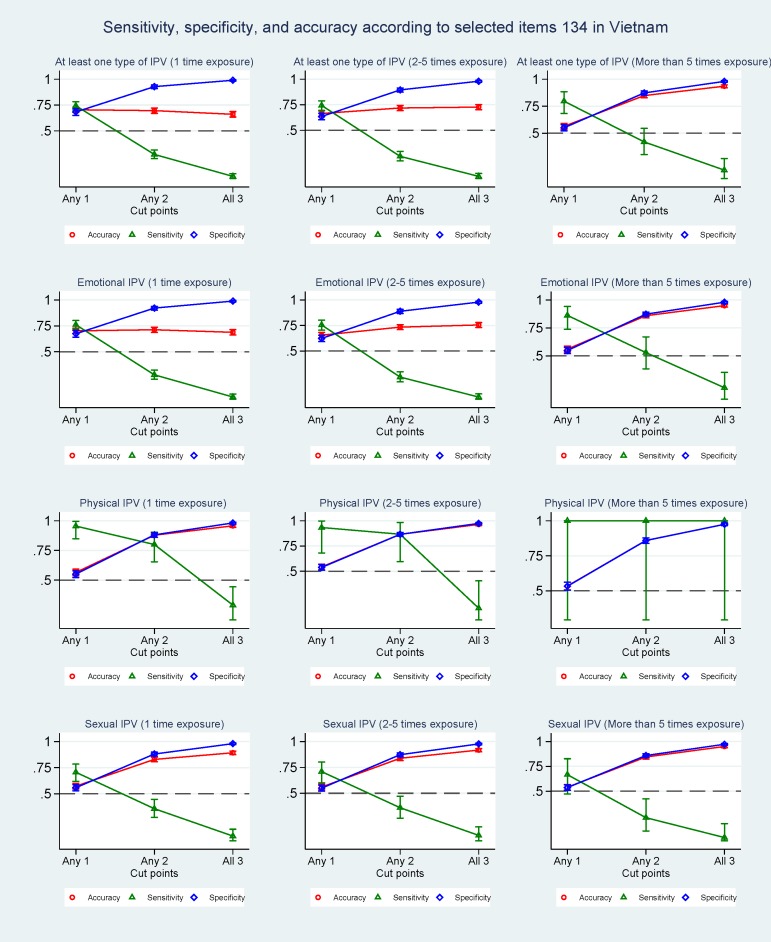
Sensitivity, specificity, and accuracy according to selected items 134 in Vietnam.

The diagnostic ability of question combination 1+3+4 in identifying IPV using the WHO tool as gold standard was tested for different cut-off values in Tanzania (Fig 2) and Vietnam (Fig 3). Using a cut-off of 2 resulted in improved specificity for detecting IPV among Tanzanian women, with values of 0.97 for at least one type of IPV, 0.96 for emotional IPV, 0.92 for physical IPV and 0.94 for sexual IPV. The accuracy also increased, with values varying from 0.93 to 0.98; it was, however, at the cost of the sensitivity, which dropped to 0.33 for at least one type of IPV, 0.38 for emotional IPV, 0.69 for physical IPV and 0.44 for sexual IPV. By using a cut-off of 3, the specificity and accuracy increased even further and the decrease in sensitivity became more pronounced. The same trend of increasing specificity and accuracy and decreasing sensitivity was found regardless of frequency of exposure. An identical picture was found in Vietnam, were a cut-off of 2 resulted in increased specificity values of 0.93 for at least one type of IPV, 0.92 for emotional IPV, 0.88 for physical IPV and 0.88 for sexual IPV, and decreased sensitivity values of 0.27 for at least one type of IPV, 0.28 for emotional IPV, 0.80 for physical IPV and 0.36 for sexual IPV. Similarly, in the Vietnamese population a cut-off of 3 resulted in a further increased specificity and accuracy and a decreased sensitivity, regardless of frequency of exposure. When comparing the three-question combination with the performance of all eight questions in predicting exposure to any type of IPV among Tanzanian women (S1 Fig), a higher sensitivity and a lower specificity for the eight question combination was found at a cut-off of 1 whereas a similar performance was found at a cut-off of 2. The picture was a bit different for Vietnam, where the performance of the eight-question combination was poorer at a cut-off of 1 and similar at a cut-off of 2 (S2 Fig).

## Discussion

A combination of three screening questions was found to be effective in identifying women exposed to IPV during pregnancy. The found 3-item combination performed best in Tanzanian whereas the sensitivity was slightly lower in Vietnam. When evaluating the test performance for the different types of IPV, the 3-item combination performed best among women who were exposed to physical IPV, with a sensitivity of 0.93 among Tanzanian women and 0.96 among Vietnamese women.

Approximately one-third of the participants in both countries had been exposed to at least one type of IPV during pregnancy. When focusing on the different types of IPV, 23% had been exposed to emotional IPV, 6.0% to physical IPV and 15% to sexual IPV in the Tanzanian setting. The corresponding Vietnamese figures were 35%, 3.5% and 10%, respectively. Globally, there is a great variation on the reported prevalence of IPV, which has been illustrated in a recent systematic review based on data from antenatal clinics, where the prevalence rates of IPV during pregnancy were found to be 2–57% (n  =  13 studies), with meta-analysis yielding an overall prevalence of 15% [20]. The large difference in reported IPV prevalence rates likely reflects that IPV is a complex phenomenon to measure where different approaches have been employed over the years [21].

We found that a screening based on the three questions with a 93% probability would be positive among Tanzanian women exposed to physical IPV and with 96% probability would be positive among Vietnamese women exposed to physical IPV. When focusing on emotional and sexual IPV, the probability dropped to 78% and 87% among Tanzanian women and to 76% and 71% among Vietnamese women. The three-question combination’s poorer performance in predicting emotional IPV may reflect that an experience defined as emotional IPV in a screening instrument is not always considered as abuse by the victim and therefore the act may not be disclosed by them. When it comes to sexual IPV, a comparatively lower sensitivity was found in the Vietnamese study setting. This shortcoming of the found item combination most likely reflect that there are a number of barriers that may hinder questioning adult women about sexual abuse as well as barriers that may hinder women from sharing such experiences. In other words, a brief screener, like the one we have developed, may not provide sufficient confidentiality to obtain valid answers on sensitive topics such as sexual activity. Other studies have likewise shown that asking about sexual IPV specifically may be even more difficult than asking about physical IPV [22]. This may mirror a general societal discomfort about sexual IPV, a misunderstanding of what sexual assault is and the taboos about directly asking questions regarding anything sexual. The three-question combination performed differently at different cut-off levels. In both Tanzania and Vietnam, a picture of increasing specificity at the cost of decreasing sensitivity was observed when the cut-off values increased from 1 to 3. The choice of a particular cut-off value is not a statistical decision; in general, there is a trade-off between sensitivity and specificity, and the decision must be based on their relative importance [23]. We consider a cut-off of 1 to be the most optimal cut-off level since we believe that additional assessment of IPV in patients who are not involved in more severe forms of IPV is preferred above missing women who are indeed involved in severe IPV.

Based on our findings we argue that antenatal care can play an important role in identifying women who are exposed to IPV. It should, however, also be stressed that screening for IPV has to go hand in hand with supportive responses and information so the women can plan for their safety. Hence, in any screening for IPV, the expenditure of resources spent on screening must be justifiable in terms of eliminating or decreasing IPV exposure and associated adverse outcome. Therefore acceptable and affordable interventions must be in place before routine IPV screening is considered implemented [24]. In the discussion of routine screening for IPV among pregnant women, it should also be taken into account that IPV screening as well as referral of IPV victims to services is controversial in many health care settings. Mainly because health care providers lack comfort in performing screening and referral, a discomfort which is often coupled with a concern that screening and referral may cause victims harm [25]. Hence, limited time and availability of on-site IPV referral resources is together with lack of knowledge and training among providers reported to be an important barrier to screening [26]. When it comes to referral, fear of losing custody over children, fear of retribution, cultural barriers, and previous negative experiences with services have been described as barriers to seeking referral among women who are exposed to IPV [26]. It has been suggested that training of providers, ensuring a respectful and trusting relationships between victims and providers, ensuring immediacy of referral, and the implementation of institutional referral policies are factors that may facilitate IPV referral [25]. However, the evidence is sporadic and further research is needed to identify the role of the individual factors in the implementation and success of IPV referral.

There are some important limitations in this study. Firstly, both the screening questions and the WHO questionnaire rely on interviews performed by health staff, which implies that the results do not automatically reflect the IPV that actually took place and there is a risk that the women may have denied or minimized the IPV they had been exposed to. Alternative ways to obtain information on IPV exposure have been evaluated and the results have been compiled in a recent systematic review. The review found that computer-assisted self-administered questionnaire lead to higher rates of IPV disclosure in comparison to both face-to-face interview and self-administered questionnaires on paper [27]. It may, however, be argued that computer-assisted self-administered questionnaire would be difficult to use in low- and middle-income settings like Tanzania and Vietnam, as it would likely lead to selection bias, since only few Tanzanian and Vietnamese women have access to computer. This problem may be solved if the women are lent a tablet while visiting the clinic and a brief introduction in how to use it. Finally, although the three-question combination demonstrated good psychometric properties in Tanzania and fairly good properties in Vietnam, these findings do not necessarily generalize to other settings. The disclosure of IPV is likely to differ in different populations and this difference may have an impact on how well the screening instrument performs [28].

## Conclusion

By asking pregnant women three simple questions, one on emotional IPV, one on physical IPV and one on sexual IPV, we were able to identify women who were exposed to IPV during pregnancy in Tanzania and Vietnam. The question combination performed best in predicting physical IPV, where it identified 93% and 96% of Tanzanian and Vietnamese women, respectively, who were exposed to physical IPV measured according to the gold standard. Based on our findings we conclude that the developed screening instrument can help identifying women exposed to IPV so relevant action can be taken to address the problem and its associated consequences. Ideally, the tool should be incorporated in the first antenatal care visit together with questions about other risk factors that are routinely asked in early pregnancy.

## Supporting information

S1 Dataset Dataset Tanzania. (SAV)

S2 Dataset Dataset Vietnam. (SAV)

S1 Fig Sensitivity, specificity, and accuracy of using all eight questions to identify any IPV in Tanzania. (TIF)

S2 Fig Sensitivity, specificity, and accuracy of using all eight questions to identify any IPV in Vietnam. (TIF)
