# Supplementary figures and images for: Intimate partner violence (IPV): The validity of an IPV screening instrument utilized among pregnant women in Tanzania and Vietnam
Source: PLoS One. 2018 Feb 1;13(2):e0190856. doi: 10.1371/journal.pone.0190856 (PMC5794062; doi:10.1371/journal.pone.0190856)

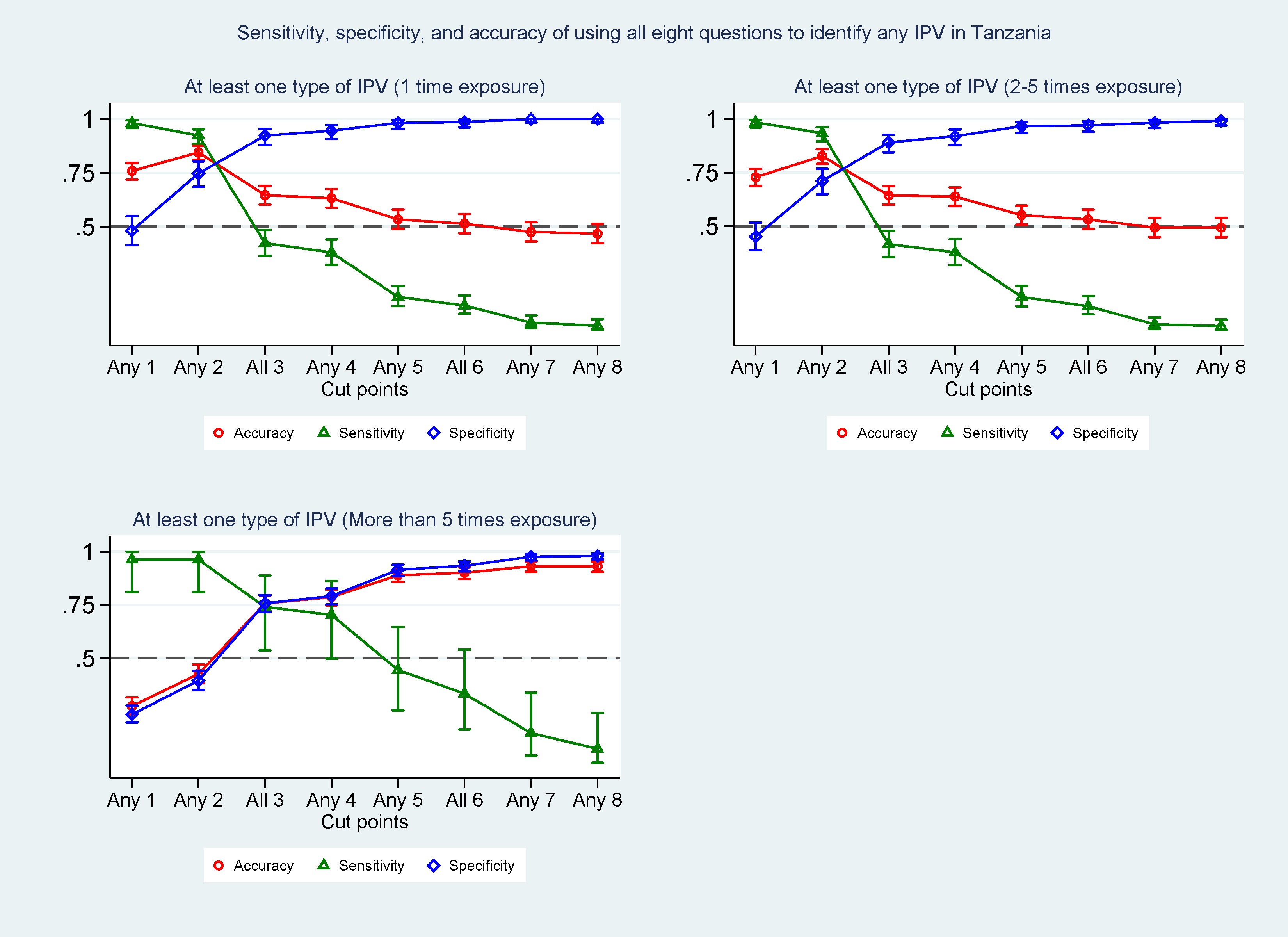

Supplement: S1 Fig — (TIF) [file pone.0190856.s003.tif]

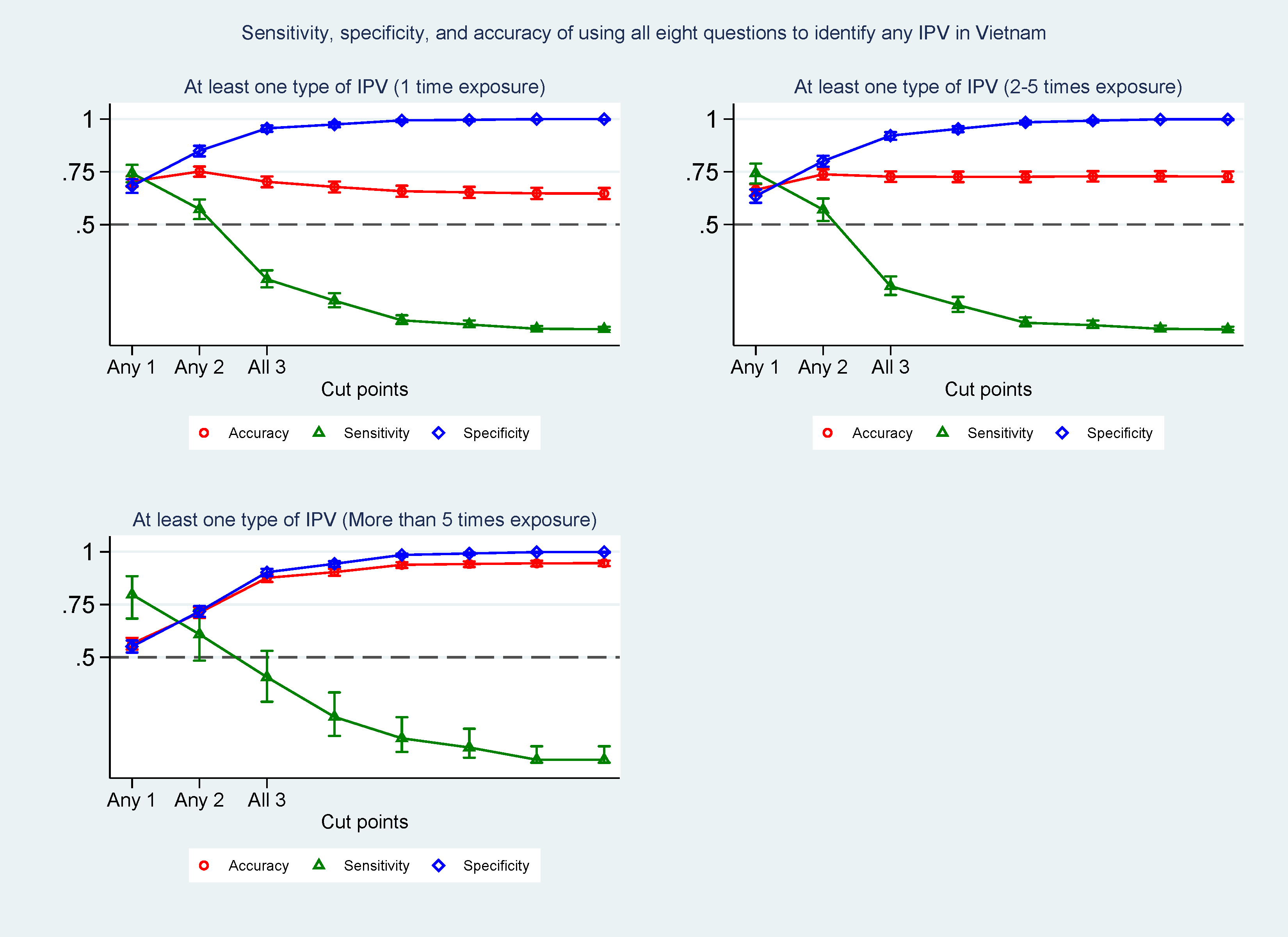

Supplement: S2 Fig — (TIF) [file pone.0190856.s004.tif]
